# Emotion regulation from a virtue perspective

**DOI:** 10.1186/s40359-023-01490-y

**Published:** 2024-01-03

**Authors:** Jeong Han Kim, Jina Chun, Jaeyoung Kim, Hyun-Ju Ju, Byung Jin Kim, Jeongwoon Jeong, Dong Hun Lee

**Affiliations:** 1 School of Rehabilitation Services and Counseling, University of Texas – Rio Grande Valley, 1201 W University Dr, Edinburg, TX 78539 USA; 2 Department of Rehabilitation Psychology and Special Education, University of Wisconsin- Madison, 1000 Bascom Mall, Madison, WI 53706 USA; 3 Department of Counseling, Educational Psychology, and Special Education, Michigan State University, 620 Farm Lane, Erickson Hall Rm. 459, East Lansing, MI 48824 USA; 4 Department of Early Childhood, Special Education, and Counselor Education, University of Kentucky, 597 S Upper St, Lexington, KY 40508 USA; 5 Department of Counselor Education and Rehabilitation, California State University-Fresno, 5241 N Maple Ave, Fresno, CA 93740 USA; 6 Department of Individual, Family, and Community Education, The University of New Mexico, Albuquerque, NM 87131 USA; 7 Traumatic Stress Center, Department of Education, Sungkyunkwan University, 51112 Hoam Hall, 25‑2, Sungkyunkwan‑ro, Jongno‑gu, 03063 Seoul, Republic of Korea; 8 Traumatic Stress Center, Department of Education, Sungkyunkwan University, 16419 Seoul, South Korea

**Keywords:** Emotion regulation, Psychosocial adaptation, Virtue, V-PAM

## Abstract

**Background:**

The ability to regulate one’s emotional state is an important predictor of several behaviors such as reframing a challenging situation to reduce anger or anxiety, concealing visible signs of sadness or fear, or focusing on reasons to feel happy or calm. This capacity is referred to as emotion regulation. Deficits in this ability can adversely affect one’s adaptive coping, thus are associated with a variety of other psychopathological symptoms, including but not limited to depression, borderline personality disorder, substance use disorders, eating disorders, and somatoform disorders.

**Methods:**

The present study examined emotion regulation in relation to the virtue-based psychosocial adaptation model (V-PAM). 595 participants were clustered based on their Difficulties in Emotion Regulation Scale (DERS) score, producing two clusters (i.e., high functioning vs. low functioning). Then, emotion regulation group membership was discriminated by using five V-PAM virtue constructs, including courage, integrity, practical wisdom, committed action, and emotional transcendence.

**Results:**

Results show that five virtues contribute to differentiating group membership. Practical wisdom was the strongest contributor, followed by integrity, emotional transcendence, committed action, and courage. Predictive discriminant analysis was conducted and 71% of cases were correctly classified. A discussion of the relationship between emotion regulation and virtues was elaborated.

**Conclusion:**

The concept of virtue holds significant importance in the comprehension of an individual’s capacity to regulate their emotions, meriting future study.

## Introduction

Emotion regulation and virtues are two important protective factors for positive psychosocial adaptation to life adversity. While the exploration of virtue is a relatively recent endeavor within the context of psychosocial adaptation, emotion regulation is well-established. Emotion regulation refers to the ability to control emotional responses and behaviors including thinking through the situation. Theoretical connections between emotional regulation and virtue emerge from perspectives in physical and behavioral medicine. Di Giacomo and colleagues [1] examined the impact of psychological aspects within the scope of hypertension management via the lens of the relationship between emotion regulation and self-care skills. Similarly, Leukel et al. [2] investigated the role of emotion regulation in relation to diabetes-related distress management. Both studies found the significant influence of emotion regulation on enhancing self-care skills and promoting improved psychological outcomes. This evidence is further corroborated by an integrative review study conducted by Wierenga, Rebecca, Lehto, and Given [3], which underscores the impact of emotion regulation on behaviors in healthy individuals.

In contemporary counseling, virtue is operationalized as the consistent practice of personally important values. Although universally valued, such as honesty, may not be consistently practiced by all people. The exploration of virtues within the areas of health and behavioral medicine has been a growing area of study. Hanks and colleagues’ research showed that virtues play a predictive role in fostering greater life satisfaction and perceived community engagement. However, a relatively weaker association was found between virtues and objective well-being [4]. Additionally, a strong relationship between the virtues of honesty and integrity and the lower risk of depression was also found in a 2021 study by Weziak-Bialowolska and colleagues [5]. Given that both emotion regulation and virtues involve behavioral action and contribute to overall well-being, a theoretical linkage between these two constructs exists. Therefore, the present study aims to identify this relationship.

### Emotion regulation and well-being

Emotion regulation involves positive and negative emotions [6]. This process of emotion regulation enables the recognition of antecedent conditions that trigger specific emotions as well as the emotion themselves, thus its process has received attention from various disciplines due to the extensive impact of emotions on human behavior and well-being. Numerous empirical studies have demonstrated that emotion regulation plays a significant role in fostering adaptive cognitive style, decision-making abilities, and behavioral intentions in response to social and situational demands, ultimately impacting an individual’s well-being [7].

The emotion regulation strategies further involve situation selection/modification (i.e., active coping), attentional deployment (i.e., distraction and rumination), cognitive change (i.e., cognitive reappraisal), and response-modulation (i.e., expressive suppression) in response to stressful situations, while emotion dysregulation often leads to maladaptive behaviors. For example, according to Folkman and Lazarus [8], two critical factors that determine an individual’s coping behaviors are their perceived appraisal of stressful situations and their own capacities to respond to these situations. When faced with stressful situations, individuals can use cognitive reappraisal or restructuring as their emotional regulation strategy to moderate their perception of the situation. This reappraisal leads to more adaptive ways of interacting with the situation. Conversely, poorly regulated emotions are associated with maladaptive cognitions such as repetitive rumination and rehearsal [9, 10]. In sum, emotion regulation can contribute to increased psychological well-being by helping individuals to evaluate and use more adaptive coping strategies to effectively address stressful situations rather than relying on maladaptive coping behaviors to avoid stressors or suppress negative emotions simply. Previous literature also has supported the beneficial impacts of emotion regulation strategies associated with solution-focused coping and elevated psychological well-being. In contrast, dysregulation has been strongly related to psychological distress and maladaptive behaviors [11, 12].

### Virtue and well-being

Virtue is an emerging area of study in the context of well-being and life thriving [13]. In the early history of psychology, it was once a popular topic, but it became disfavored with the rise of empiricism in psychological science. This was due to the perception that virtue was more moral and philosophical concepts, making them difficult to investigate empirically [14]. However, the importance of virtue in behavior science was reclaimed with the growth of positive psychology. It was because founders of positive psychologists such as Peterson and Seligman were able to provide theoretical definitions of virtues from a character perspective [13, 14]. This was achieved by distinguishing virtue from value. Value is what an individual thinks is important, while virtue is the value a person practices. For example, while many people may consider honesty to be important, not everybody lives upon the value of honesty. When a person constantly practices honesty or other values, it becomes integrated into their character that represents who the individual is. Thus, the value of honesty is difficult to measure. But, when it became one’s character through the constant practice of honesty, it becomes measurable in modern psychology. In that, a character is a behavior manifestation of virtue. This operational distinction between virtue and value enabled empirical investigation of virtue. This theoretical distinction has also had a significant impact on the counseling field as it sparked an empirical investigation of the relationship between virtue and life-thriving. The following section highlights some of the recent research evidence in this area.

Positive psychology study conducted in the early 21st century found that hope, zest, love, gratitude, and curiosity were to be substantially related to life satisfaction, while modesty, appreciation of beauty, creativity, judgment, and love of learning were found to have a weaker association [15]. Peterson and colleagues [16] extended their study to examine the relationship between character traits and recovery from illness. They found that individuals who had recovered from a serious illness exhibited higher levels of appreciation of beauty, bravery, curiosity, fairness, forgiveness, gratitude, humor, kindness, love of learning, and spirituality compared to those who had never experienced such an illness. In 2009, Park and Peterson [17] replicated their study with a young population and found love, gratitude, hope, and zest are relevant to adolescents’ life satisfaction. In 2011, Proyer and colleagues [18] reconfirmed that hope, gratitude, love, zest, and curiosity are important in life satisfaction, and hope and spirituality were the strongest predictors of life satisfaction. In 2016 and 2018, Kim and colleagues [19, 20] also conducted research on whether virtue could differentiate adaptation and resilience levels following the onset of disability. The finding was that one’s long-term commitment and emotional transcendence were the most significant contributors in differentiating their adaptation to chronic illness and disability.

### Purpose and research questions

Given the role of emotions and virtue in regard to the individuals’ life and well-being, it is important to consider how a person’s ability to effectively manage and respond to an emotional experience, known as emotion regulation, is related and integral to the development and maintenance of virtues. However, few studies have conducted the relationship between virtues/character traits and emotion regulation. Thus, the purpose of this study was to examine the role of virtues in emotion regulation. The following research questions were of interest of the current study:

1. Is it possible to categorize participants into two or more groups that reflect one’s ability to regulate their emotion, conceptualized in terms of difficulties in emotion regulation? Then, what are the unique group characteristics?
2. Do virtues have a contributory effect in differentiating participants’ group membership determined based on their ability to regulate emotion?

## Method

### Participants and procedure

A total of 845 Koreans aged between 20 and 59 participated in the present study with 595 cases being retained after data-cleaning procedures. The survey took approximately 30 min to complete, and the participants received online credit points as compensation. The online survey was conducted using a secure internet-based survey company, which utilizes a firewall (WAF) and the DigiCert security service, a reputable digital certificate association. The online survey system is regularly scanned for vulnerabilities by automated systems that network environment, operating system, is periodically tested. Additionally, all survey responses are collected through encrypted Secure Sockets Layer connections (SSL: the standard technology for establishing an encrypted link between a web server and a browser), ensuring the security of private information. After the survey is completed, the company ensures that all data was securely erased and destroyed.

Among 595 adults who participated in the research, 325 (54%) were male and 270 (44%) were female. The largest age group was individuals in their 40s (31%), followed by those in their 30s (28%), 20s (21%), and 50s (20%). The majority of participants were heterosexual (494, 83%) characteristics with middle (380, 64%) and upper middle class (138, 23%). When asked about self-identified mental illness (i.e., ever hospitalized or consider hospitalization due to mental illness), most participants answered no (520, 87%). Of the total sample, 106 (18%) indicated having a chronic illness or a disability (CID). CID in the present study refers to any illness that lasts over a 6-month period. Among those 106 people with CID, 93 individuals (83%) were receiving disability related benefits from South Korean government. Other disability related characteristics were summarized in Table 1.

**Table 1:** Sample Characteristics

	Frequency	Percent		Frequency	Percent
**Gender**			Disability Benefit		
Male	325	55	Yes	13	12
Female	270	45	No	93	88
Total	595	100	Total	106	100
**Age**			**Disability Type**		
20–29	125	21	Acquired	5	5
30–39	164	28	Congenital	101	95
40–49	185	31	Total	106	100
50–59	121	20			
Total	595	100	**Functional Limitation**		
			Mobility	28	26
**Sexual Orientation**			Communication	3	3
Heterosexual	495	83	Self-Care	11	10
LGBTQ++	12	2	Self-Direction	11	10
Prefer not to answer	88	15	Interpersonal Skills	22	21
Total	595	100	Work Tolerance	27	26
			Work Skills	4	4
**SES**			Total	106	106
Poor	75	13			
Middle	380	64	**Perceived Severity - Physical Health**		
Upper Middle	138	23	Mild	51	48
Affluent	2	3	Moderate	38	36
Total	595	100	Severe	17	16
			Total	106	100
**Mental Illness (Self-Identified)**					
Yes	75	13			
No	520	87	**Perceived Severity - Mental Health**		
	595	100	Mild	56	53
			Moderate	29	27
**Chronic Illness and Disability**			Severe	21	20
Yes	106	17.8	Total	106	100
No	489	82.2			

*Note.* SES: Socioeconomic Status

### Instruments

#### Difficulties in emotion regulation scale

Difficulties in Emotional Regulation Scale (DERS) [21] translated by Cho [22] was used to operationalize participants’ ability to regulate their emotions. DERS includes 36 items designed to ask respondents how they relate to their emotions to produce scores on six subscales: (1) nonacceptance of emotional responses, (2) difficulty engaging in goal-directed behavior, (3) impulse control difficulties, (4) lack of emotional awareness, (5) limited access to emotion regulation strategies, and (6) lack of emotional clarity. DERS utilizes a five-point rating scale (*1 = almost never, 2 = sometimes, 3 = about half the time, 4 = most of the time, and 5 = almost always*), with a higher score indicating more difficulties. Based on a sample of 427 adults presenting at an outpatient clinic diagnosed with one or more DSM-5 disorders, Hallion and colleagues [23] found that the DERS had good internal consistency, particularly when the Awareness subscale is excluded. This indicates that Awareness may be a separate construct. Thus, in the Korean version of DERS, the Awareness subscale was not included.

### Adapted inventory of virtues and strengths

Adapted Inventory of Virtues and Strengths (AIVS) [20] is a 46-item measure with five subscales that operationalizes five virtue factors of V-PAM (i.e., courage, integrity, practical wisdom, committed action, and emotional transcendence) in terms of corresponding character traits. Utilizing a 7-point semantic differential scale with pairs of opposing adjectives or short phrases anchoring the two extremes, AIVS can be administered in 10–15 min. To reduce response bias, the polarities of some items are reversed and the higher score of each item indicates a positive self-perception of the testing item (e.g., coward – bravery). Reported alphas of AIVS in previous studies range from 0.70 to 0.90, indicating acceptable to strong reliability [20, 24, 25]. The alpha coefficients of AIVS factors in the current samples ranged from 0.77 to 0.86.

### Data analysis

Cluster analysis, analysis of variance (ANOVA), and discriminant analysis were employed for the current study. Cluster analysis was used to divide the sample into smaller subgroups, based on the levels of difficulties in emotional regulation. Then, ANOVA was performed to examine the distinct characteristics in terms of emotional regulation between the subgroups, identified from the cluster analysis. Finally, discriminant analysis was conducted to identify the contributory effects of five virtue subconstructs on group membership.

## Results

### Cluster analysis followed by ANOVA

**Fig. 1:**
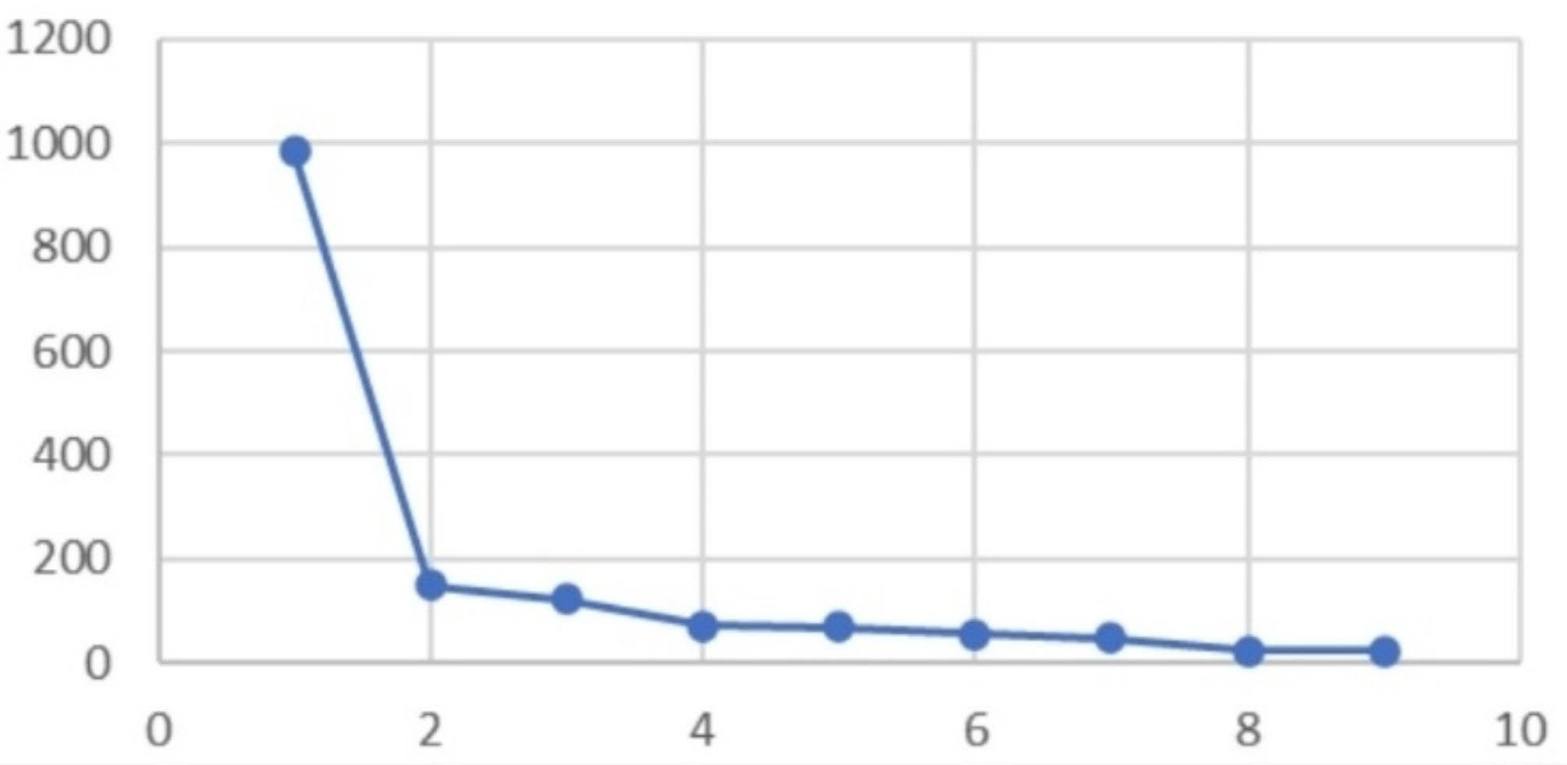
Scree test

To address research question 1, cluster analysis followed by ANOVA was employed. To determine the proper cluster solution, changes in agglomeration coefficient and scree plot were inspected. Concerning changes in agglomeration coefficients (Table 2), there was a significant change from 1 cluster to 2 clusters, but small changes after 2 cluster solutions. This suggests the two-cluster solution. The elbow of the scree plot (Fig. 1) was also examined, confirming that 2-cluster solution was optimal. To identify group characteristics, a univariate analysis of variance was followed (Table 3). Group 1 was showing higher scores in all DERS subscales and Group 2 was showing lower scores on all DERS subscales (i.e., impulse control difficulties, nonacceptance of emotional response, lack of emotional clarity, limited access to emotion regulation strategies, difficulty engaging in goal-directed behavior). Group 1 was labeled as lower functioning in emotion regulation (i.e., higher score indicating more difficulties in emotion regulation), while Group 2 was labeled as higher functioning in emotion regulation.

**Table 2:** Changed in Agglomeration Coefficient

# of Cluster	Agglomeration Coefficients	Changes
10	546	23
9	569	23
8	592	48
7	640	56
6	696	68
5	764	71
4	835	129
3	964	148
2	1112	984
1	2096	

**Table 3:** ANOVA table

	Group	N	Mean	Std. Deviation	F Statistics
DERS_ICD	1	343	3.0748	0.49221	*F*(1, 593) = 445.646**
	2	252	2.1362	0.5902	
DERS_NER	1	343	3.0875	0.5336	*F*(1, 593) = 910.184**
	2	252	1.6759	0.60278	
DERS_LEC	1	343	2.9461	0.65951	*F*(1, 593) = 412.753**
	2	252	1.7758	0.73902	
DERS_LAERS	1	343	3.0309	0.53012	*F*(1, 593) = 760.395**
	2	252	1.7659	0.58257	
DERS_DEGB	1	343	3.1701	0.62119	*F*(1, 593) = 278.667**
	2	252	2.2077	0.78419	
V_ET	1	343	4.0957	0.56844	*F*(1, 593) = 94.397**
	2	252	4.5992	0.69381	
V_PW	1	343	4.0676	0.64308	*F*(1, 593) = 98.269**
	2	252	4.6468	0.77996	
V_IT	1	343	4.4278	0.70711	*F*(1, 593) = 96.455**
	2	252	5.0256	0.76842	
V_CO	1	343	3.7918	0.78132	*F*(1, 593) = 27.328**
	2	252	4.2016	1.12996	
V_CA	1	343	4.1653	0.76952	*F*(1, 593) = 63.481**
	2	252	4.7103	0.89377	

*Note.* DERS_ICD: Impulse Control Difficulties; DERS_NER: Nonacceptance of Emotional Response; DERS_LEC: Lack of Emotional Clarity; DERS_LAERS: Limited Access to Emotion Regulation Strategies; DERS_DEGB: Difficulty Engaging in Goal-Directed Behavior; V_ET: Emotional Transcendence; V_PW: Practical Wisdom; V_IT: Integrity; V_CO: Courage; V_CA: Committed Action

### Discriminant analysis

To examine the contributory effect of virtue in differentiating emotion regulation levels (research question 2), group memberships (lower functioning vs. higher functioning) were discriminated by five V-PAM virtues. And, λ = 0.811, F (5, 590) = 123.51 *p* = .00, indicating virtue contributes to differentiating emotion regulation membership. Structure matrix that shows the correlation between discriminant variables and discriminant function revealed that Practical Wisdom was the strongest predictor (0.844) followed by Integrity (0.836), Emotional Transcendence (0.827), Committed Action (0.678), and Courage (0.445). Utilizing predictive discriminant analysis, group members of the original case were predicted and 71% of original cases were correctly classified. The results of discriminant analysis are summarized in Table 4.

**Table 4:** Discriminant Analysis

Wilks’ Lambda		λ = 0.811, F(5, 590) = 123.51 p = .00			
**Structure Matric**			**Function 1**		
Practical Wisdom			0.844		
Integrity			0.836		
Emotional Transcendence			0.827		
Committed Action			0.678		
Courage			0.445		
**Predictive Discriminant Analysis**		Ward Method	Predicted Group Membership		Total
			1	2	
Original	Count	1	286	57	343
		2	116	136	252
	%	1	83.4	16.6	100
		2	46	54	100
Cross-validated	Count	1	286	57	343
		2	119	133	252
	%	1	83.4	16.6	100
		2	47.2	52.8	100

a. 70.9% of original grouped cases correctly classified

b. Cross validation is done only for those cases in the analysis. In cross validation, each case is classified by the functions derived from all cases other than that case

c. 70.4% of cross-validated grouped cases correctly classified

b. Cross validation is done only for those cases in the analysis. In cross validation, each case is classified by the functions derived from all cases other than that case

## Discussion

In light of the fact that both emotion regulation and virtue are considered to be protective factors for enhancing human flourishing, theoretical linkage exists between the two constructs. However, limited literature indicates the needs of scientific examination of two constructs and it was the aim of the present study. Emotion regulation was operationalized in terms of impulse control difficulties, nonacceptance of emotional response, lack of emotional clarity, limited access to emotion regulation, difficulty engaging in goal-directed behavior. 595 adults were first classified into low- and high-functioning emotional regulation groups. Then, group memberships were discriminated based on five virtues. Results revealed that practical wisdom is the greatest contributor in differentiating emotion regulation functioning (0.844), followed by integrity (0.836), emotional transcendence (0.827), committed action (0.678), and courage (0.445). Discussions on the relationship between emotion regulation and each of virtue construct were elaborated.

### Practical wisdom and emotion regulation

Practical Wisdom, a construct of the five V-PAM virtues, was found to be the most significant contributor to individuals’ ability to regulate their emotions and determine group membership. Practical wisdom has been conceptualized as a set of skills that involves making best situational decisions based on knowledge, experience, and moral virtues [19, 26]. In addition, practical wisdom is linked to self-awareness and one’s ability to set situationally realistic goals [19]. Individuals with practical wisdom are adept at evaluating their strengths and limitations, identifying areas for improvement, and establishing goals that align with their values and interests. They are also capable of adapting to changing circumstances and making informed decisions based on their knowledge and experiences. Practical wisdom encompasses several components, including self-reflection, decisiveness, acceptance of uncertainty, empathy, and compassion [26]. Notably, emotion regulation is considered as one of these components and the relationship between emotion regulation and practical wisdom has been documented in various studies [27–30]. Practicing practical wisdom enables individuals to make appropriate situational decisions, especially when faced with emotionally-charged life circumstances, and helps ensure that emotional responses are not excessive or misleading [14, 27]. As such, individuals who can reevaluate their emotional experiences or take constructive measures to handle difficult situations are more adept at adjusting to life’s fluctuations [31, 32]. Taken together, based on our findings on the differential impact of virtue on emotion regulation, it is evident that practical wisdom is vital for individuals to navigate through the challenges of life and cultivate the ability to control their emotional state. Therefore, cultivating practical wisdom could facilitate positive adaptation to life adversity.

### Integrity and emotion regulation

The present study found integrity was the second most significant virtue factor that influences one’s ability to regulate emotion. This finding is relatively new in current literature, as there has been a lack of research on the direct relationship between integrity and emotion regulation. However, there exist studies that explored these two constructs from a social and relational factors associated one’s well-being. For instance, integrity is a critical factor of trust, commitment, and honesty pertaining to relational aspects of one’s life, and it is associated with the individual’s ability to manage and maintain individual relationships [33]. According to V-PAM, integrity is a communal aspect of virtue that is highly related to ability to build trust with others. Thus, integrity influences one’s inter- and intrapersonal growth, and helps individuals to be mindful in their commitment and honest in their decision-making process [25]. Therefore, the current study provides an evidence that individuals with a high level of integrity have an ability to better establish and maintain mutual relationships and support network, thus, in turn, it positively promotes one’s ability to regulate and manage emotions adaptively [34–36]. This finding aligns with previous studies that have highlighted the importance of social factors in shaping emotion regulation processes, warranting future research that examines the relationship between integrity and emotion regulation.

### Transcendence and emotion regulation

The current study confirmed that transcendence had the third contributory factor to emotional regulation. In V-PAM, transcendence is conceptualized in terms of character traits like loving, meaningfulness of life, forgiveness, optimism, thankful/gratefulness, enthusiasm, caring, and spirituality [25]. Cultivating these characters might act as a buffer against cognitive vulnerabilities that can exacerbate negative psychological symptoms [37]. From this perspective, transcendence allows an individual’s experience of psychological adversity to be understood as a stage of growth and to transform that situation into a positive perspective, such as by bringing hope into emotional challenges [13]. These traits also have been shown in the relationship with emotional regulation. For example, having a religion leads to greater emotional regulation [38], and gratitude is an effective emotion-regulation intervention that involves tuning in to what works well in our environments and savoring the positive [39]. The relationship between transcendence and regulation in the emotional aspect can also bring benefits that extend the autonomy of the client as it promotes self-reflection and further allows an individual to redefine their life experience from more constructive lens. Self-reflection is one’s ability to examine, concentrate on, and assess one’s cognitive, emotional, and behavioral processes, as well as the individual’s desire to gain a deeper understanding of their goals, purposes, and authentic self [40]. Through this reflective transcendence mechanism, individuals can navigate complex emotional situations, discerning the differences between subjective and objective aspects of life circumstance. Therefore, promoting emotional transcendence can assist people in autonomously recognizing, using, and evaluating relevant their character assets to overcome challenging situations [13].

### Commitment and emotion regulation

Adversity comes in all different forms for people in life. Personal experiences might include things like disease, the death of a loved one, abuse, acquiring a disability or illness, losing a job, and financial difficulty. Tragic news stories about terrorist attacks, mass shootings, and natural disasters are a common reality. Humans must learn how to deal with and get through difficult life circumstances. Yadav and Hooda [41], with 147 osteoarthritis patients, examined the relationship between resilience and psychological flexibility. Psychological flexibility in their study was conceptualized in terms of pain acceptance, activity engagement, pain willingness, values, mindfulness, cognitive fusion, decentering, rumination, self as context, and committed action. Results showed that pain acceptance, values, decentering, self as context, and committed action had a significant positive correlation to pain-related resilience while cognitive fusion and rumination had a significant negative relationship with resilience.

Committed action refers to a person’s long-term striving or deliberate action to overcome any difficulties in order to achieve goals, even in the face of obstacles [33]. This long-term pursuit of excellence is a key factor that promotes ultimate psychosocial adaptation to life adversity as it helps individual maintain lofty goals driven by intrinsic interests and motivations [33]. Motivation is the foundation for human learning and development [42]. Human can be motivated by various extrinsic and intrinsic factors, but intrinsic motivation is the most optimal form of motivation as it is associated with various benefits such as personal enjoyment, psychological well-being, and the promotion of behavior [43, 44]. Committed long-term action is a behavior manifestation of one’s motivation, but, most of time, it is difficult as challenging life situations will turn down individual’s motivation at any time. In such a situation, strong commitment helps an individual manage, regulate, and modulate one’s emotions to be able to adapt to different situations and environments.

### Courage and emotion regulation

Current findings support that courage plays a significant role in emotion regulation. While limited empirical studies have investigated the relationship between courage and emotion regulation directly, existing literature on courage in relation to resilience provides valuable insight. Courageous individuals willingly confront and actively cope with life difficulties to achieve their goals in stressful situations, and the ability to regulate emotions in such situations is critical for maintaining resilience. Therefore, courage can be a powerful tool in helping individuals regulate their emotions, and being courageous in the face of adversity requires emotional regulation to respond to challenging life circumstances in an adaptive manner.

Courage in counseling is often discussed in the context of fear-related emotion regulation. From Adler perspective, fear is central to inferiority and courage is the essential component required to overcome inferiority [45]. Adler [46] states that “a good compensation will be made only where there are courage (p.16)…, by courage, disabilities may be so compensated that they even become great abilities (p.44).” Recent research supports the idea that courage plays a significant role in psychological well-being through emotion regulation. Magnano et al. [47] found that people with higher levels of courage are better able to manage emotions like fear and use greater coping skills, contributing to psychological well-being. Courage is also associated with life satisfaction [48], elevated psychological capitals such as optimism, hope, and resilience [49, 50], and reduced psychological distress [51]. Furthermore, with courage, individuals can acknowledge difficult emotions such as fear and anxiety and allow them to confront, regulate, and process these emotions more effectively, instead of suppressing or avoiding them. In disability and health-related study, courage helps individuals cope with chronic illness and disability and overcome destructive habits as people gain the necessary power to execute their will despite the fears associated with CID [13]. In a nursing-related study, Norton and Weiss [52], courage is a trait that enables a nurse to regulate fear in a high-risk emergency that requires fast decisions critical to one’s life [53]. In a study on anxiety, courage, and resilience among nursing students caring for COVID-19 patients, the researchers found a significant and indirect correlation between courage and resiliency [54].

### Counseling implications

The concept of emotion regulation encompasses cognitive and attentional processes that facilitate the modulation of emotional states in response to life challenges. Effective emotion regulation strategies may vary depending on the situation and can be adaptive or maladaptive for individuals. In the context of health-related counseling, it is evident that emotional dysregulation contributes to reduced mental health and overall quality of life in the face of life stressors. Factors such as instability in emotion and behavioral regulation, interpersonal relationships, and self-image have been identified as key contributors [55]. Furthermore, research indicates that maladaptive emotion regulation strategies may increase the risk of chronic illness, such as chronic pain [56–58]. Notably, maladaptive emotion regulation patterns are often observed in individuals on the autism spectrum who have comorbid anxiety due to greater nonacceptance of emotions and difficulties with goal-directed behaviors [59].

The integration of emotion regulation strategies, including situational selection/modification, active emotion change, emotion acceptance, attentional deployment, and emotional resilience, emerges as a valuable approach in assisting individuals to manage symptoms of disabilities. These strategies prove beneficial in coping with negative emotions and stress associated with the adaptation process. A randomized controlled trial highlighted the efficacy of combined exposure therapy with emotion-regulation techniques for those with comorbid chronic pain and emotional issues. This approach demonstrated greater effectiveness in reducing pain-related negative thoughts, depression, and the impact of pain on daily activities compared to a Cognitive Behavioral Therapy (CBT)-based guided Internet pain management program [60]. Moreover, the integration of emotion regulation with virtue appears to facilitate the adaptation process by increasing cognitive diffusion, informed situational decisions, goal-oriented behavior, and committed action. This holistic approach acknowledges the interconnectedness of emotion regulation and virtues in promoting overall well-being and adaptive coping mechanisms.

The strong association of emotion regulation with the use of adaptive coping strategy provides important insight into the study of psychosocial adaptation to CID. Having CID often brings a unique task to shape an individual’s perspective on themselves, their health conditions, and their way of interacting with environments in the context of disability. Also, it often leads to great changes across a variety of life domains and is frequently associated with a societal stigma and disadvantages. Consequently, the process of psychosocial adaptation to CID can be a demanding and stressful process that requires major emotional regulation and coping capacity to adjust one’s attitudes, values, roles, and goals in changing environments due to CID. In this regard, scholars have highlighted the importance of coping in psychosocial adaptation [61, 62]. Empirical research also has focused on the role of emotion regulation in coping behaviors and provided valuable implications on how emotion regulation is associated with adaptive coping, resilience, increased psychosocial functioning, and well-being [63, 64]. The current findings add empirical value to the body of psychosocial adaptation research by identifying how emotional regulation is associated with virtue.

In a recent qualitative study by McGee and colleagues [65], the expression of virtues among individuals in the early stages of dementia was examined, highlighting a gap in the literature regarding interventions that leverage these factors to address the psychological well-being of persons living with dementia. The study’s findings suggest that virtues such as transcendence, wisdom, and courage play essential roles in improving psychosocial well-being. Within the framework of transcendence, religious faith was found to assist participants in navigating difficult life circumstances and overcoming devastating emotions. Additionally, participants viewed the early stage of dementia as a positive opportunity for early intervention and management due to their awareness of the heightened risk of developing the disease with age. Finally, participants encouraged themselves by actively engaging in programs that provided specific cognitive exercises. Overall, virtues can assist individuals in finding meaning and purpose in their lives, which they may have been searching for [65].

Counselors are able to better support people with disabilities on emotion regulation by understanding their virtue and related strengths. Since individuals tend to show different levels of ability to regulate their emotions based on their virtues, counselors may examine clients’ virtue to understand how such virtue influences their emotion regulation, which is closely related to their well-being. For example, counselors can provide interventions by assessing how clients have established their virtue (e.g., practical wisdom, integrity) and how they currently perceive their virtues influencing emotion regulation. Such exploration may promote the clients to develop their virtues as strengths. In addition, counselors can offer more tailored interventions on how to better regulate emotion by understanding clients’ virtue.

Emotion regulation can be viewed from V-PAM transcendence perspective. Emotional transcendence is a construct that describes the ability to rise above difficult circumstances and experience positive emotions despite negative life events. Multiple sclerosis (MS), a neurological disorder, can have a significant impact on a person’s emotional well-being, leading to symptoms such as anxiety, depression, and mood swings. Individuals with MS may face a range of challenges and stressors related to the disease, including physical symptoms, social isolation, and uncertainty about the future. Emotional transcendence may allow individuals with MS to cultivate a sense of hope, resilience, and positive emotion, even in the face of these challenges. Some research has suggested that mindfulness-based interventions, which involve cultivating present-moment awareness and non-judgmental acceptance of thoughts and emotions, may be helpful for individuals with MS in developing emotional transcendence and improving emotional well-being. Additionally, social support and other forms of therapy may also be beneficial in promoting emotional transcendence and reducing symptoms of anxiety and depression [66, 67]. Although more research is needed to fully understand the relationship between emotional transcendence and emotional regulation, findings of the present study warrant future study.

The present research suggests that virtue may serve as a crucial factor for intervention in promoting the psychosocial adaptation of individuals with disabilities who face challenges and difficulties due to their disability status. Previous research has shown that lack of emotional regulation is a significant predictor of maladaptive reactions to life adversities, including post-traumatic symptoms, addictive behaviors, and clinical mental health symptoms. In this regard, the current study proposes that practitioners can help consumers develop resilience by focusing on the various virtues within themselves. Practitioners can use standardized assessment tools, such as the AIVS [20], to identify clients’ virtue profiles and provide psychosocial education on how each virtue can enhance emotional regulation. Engaging consumers in discussions about how their virtues can be used to overcome challenges provides a valuable opportunity for them to take an active role in their psychosocial adaptation process. In summary, the current research findings may inform the practitioners that paying attention to consumers’ virtues in connection with emotional regulation could potentially advance the implementation of an empowering and strength-based service approach to facilitate the psychosocial adaptation of individuals with disabilities.

## Conclusion

The present study examined emotion regulation from V-PAM perspective. V-PAM explains one’s adaptation to life adversity in terms of five virtues, including courage (i.e., ability to execute will), integrity (i.e., ability to hold on to values they think important but help an individual build trust with others), practical wisdom (i.e., ability to make a best situational decision), committed action (i.e., ability to deliver long-term constant action), and emotional transcendence (i.e., ability to transform adversity experience into insight and renewal). In comparison to previous findings in the V-PAM study, the current study proposes an interesting finding relevant to the counseling field. When V-PAM was examined in relation to resilience and adaptation to chronic illness and disability, committed action and emotional transcendence are often identified as the strongest contributors that distinguish resilience and adaptation level. Then, the contributory virtue effect may vary depending on sample characteristics. For example, integrity, a measure of social connectedness in V-PAM, was an important contributor to adolescents’ well-being while courage was more important than practical wisdom and integrity in a sample of college students [68]. However, the present study revealed that, when V-PAM was applied in the context of one’s ability to control emotion, practical wisdom, integrity, and emotional transcendence were stronger contributors than committed action and courage in differentiating emotion regulation. In that, practical wisdom and transcendence are cognitive and emotional factors and integrity is relational factor associated with life thriving in V-PAM, the results warrant future study concerning relative importance of cognitive and social aspects of virtues in regulating one’s emotion.

## Data Availability

The data used in the present study are available from the corresponding author upon reasonable request.
